# Cost-effectiveness of health care service delivery interventions in low and middle income countries: a systematic review

**DOI:** 10.1186/s41256-018-0073-z

**Published:** 2018-06-08

**Authors:** Samuel I. Watson, Harvir Sahota, Celia A. Taylor, Yen-Fu Chen, Richard J. Lilford

**Affiliations:** Warwick Medical School, University of Warwick, Coventry, CV4 7AL UK

**Keywords:** Health service delivery, Economic evaluation, Service delivery intervention, Cost-effectiveness, Systematic review

## Abstract

**Background:**

Low and middle income countries (LMICs) face severe resource limitations but the highest burden of disease. There is a growing evidence base on effective and cost-effective interventions for these diseases. However, questions remain about the most cost-effective method of delivery for these interventions. We aimed to review the scope, quality, and findings of economic evaluations of service delivery interventions in LMICs.

**Methods:**

We searched PUBMED, MEDLINE, EconLit, and NHS EED for studies published between 1st January 2000 and 30th October 2016 with no language restrictions. We included all economic evaluations that reported incremental costs and benefits or summary measures of the two such as an incremental cost effectiveness ratio. Studies were grouped by both disease area and outcome measure and permutation plots were completed for similar interventions. Quality was judged by the Drummond checklist.

**Results:**

Overall, 3818 potentially relevant abstracts were identified of which 101 studies were selected for full text review. Thirty-seven studies were included in the final review. Twenty-three studies reported on interventions we classed as “changing by whom and where care was provided”, specifically interventions that entailed task-shifting from doctors to nurses or community health workers or from facilities into the community. Evidence suggests this type of intervention is likely to be cost-effective or cost-saving. Nine studies reported on quality improvement initiatives, which were generally found to be cost-effective. Quality and methods differed widely limiting comparability of the studies and findings.

**Conclusions:**

There is significant heterogeneity in the literature, both methodologically and in quality. This renders further comparisons difficult and limits the utility of the available evidence to decision makers.

## Background

Low and middle income countries (LMIC) face the highest burden of disease which they must tackle despite severe resource limitations [1]. A key aspect of this is ensuring that health services and the delivery of health care are run in the most efficient way possible. This includes deployment of the least expensive human resources compatible with competent practice, for example. However, the majority of economic evaluation evidence concerns high income countries, [2] which is unlikely to be generalizable to LMICs [3]. Furthermore, while methods for the economic evaluation of patient-level clinical interventions such as specific pharmaceuticals have been well-developed and embedded into health technology assessment in high income countries [4], very limited evidence exists for service delivery interventions. And again this mainly concerns high income countries [5, 6].

A service delivery intervention can be conceived as sitting between clinical interventions acting directly on the patient and national or regional level policy interventions [5]. More specifically, service delivery interventions affect: to whom care is provided, and by whom; where care is provided; information, communication and technology that enhance the care process; and improvements to the quality and safety of care. Specific service delivery interventions in LMICs include: task shifting from more highly trained healthcare professionals to other health workers with less training and fewer qualifications, such as community health workers (CHWs); use of secondary care providers in guideline dissemination to change outpatient referral rates; outreach clinics, such as provision of a night clinic for sex workers; and emergency care quality improvement training [7]. We exclude financing and governance arrangements, which are considered policy-level interventions.

A number of previous and ongoing attempts have been made to review and rank clinical interventions in LMICs by cost-effectiveness [8–10]. A notable example is the World Bank’s *Disease Control Priorities in Developing Countries*, which aims to identify the most effective and cost-effective interventions to tackle a wide range of disease areas across LMICs. The discussion of cost-effectiveness in these analyses is typically with respect to whether or not to provide an intervention or treatment. For example, provision of anti-retroviral therapy to prevent mother-to-child transmission of HIV/AIDS or isoniazid treatment for TB are widely considered cost-effective when the comparator is no treatment [10]. However, knowledge remains limited regarding the most cost-effective way to organise the delivery of these interventions.

There is a growing volume of literature assessing CHW programs. The use of CHWs has proven to be an important service delivery innovation in low income countries as they can provide basic health services at a low cost. Recent systematic reviews of economic evaluations of CHW programs have identified a number of relevant studies [11, 12]. However, they concluded that these were generally for the introduction of small, vertical programs and not more relevant large scale, horizontally-focussed initiatives. From an economic perspective many of these studies were furthermore of limited utility, because they did not include a measure that integrated estimates of incremental costs and incremental benefits, such as the expected net benefits or incremental cost-effectiveness ratio (ICER), which is required to inform resource allocation and service delivery decisions [13]. The review by Nkonki and colleagues incorporated many studies that reported only costs or average costs and average benefits [11]. There remains a question around the evidence from ‘full’ economic evaluations for service delivery interventions, including those for CHW programmes.

In 2008, Lewin et al. conducted an overview of the evidence of the effectiveness of service delivery interventions in LMICs with the aim of “supporting the delivery of cost-effective interventions.” They organised these interventions into three categories: delivery arrangements, governance arrangements, and financial arrangements [7]. At the time the authors identified a lack of high quality evidence in this area and did not consider whether the interventions themselves were indeed cost-effective. However, they provided a useful taxonomy and definition of service delivery interventions, which we make use of here to examine the published evidence on their incremental cost-effectiveness or cost-benefit.

We aimed to review the use of health economic evaluation for service delivery interventions in LMICs. There is often considerable heterogeneity in study design between health economic evaluations, even for the same intervention. Various forms of effectiveness evidence may be incorporated, a wide variety of models may be used to extrapolate to the endpoints of interest, and the endpoints themselves may vary from disease specific outcomes, such as cases of tuberculosis treated, to generic outcomes, such as disability adjusted life years (DALY) averted. These differences may make it difficult for a decision-maker to discern which evidence is relevant to them. There is therefore a strong need to conduct systematic searches of studies in this area and amalgamate and review them. The aims of this article are fourfold. First, we aim to describe the scope of economic evaluations in service delivery research in LMICs. Second, to describe the quality of the studies and to compare and contrast methods used. Third, to summarise as far as possible the findings from the studies. And fourth, to make recommendations for policy and practice.

## Methods

We conducted a systematic review of economic evaluations of service delivery interventions in LMICs.

### Definition of a service delivery intervention

There exists no universally accepted definition of a service delivery intervention, which we discern to lie between clinical interventions and policy interventions in their proximity to the patient. Acknowledging inevitable overlap between intervention types, we considered (for the purpose of this review) service delivery interventions as those that could be judged as being classified in the taxonomy specified by Lewin et al. as affecting ‘Delivery arrangements’, but not financial and governance arrangements, which were considered policy interventions [7]. Specifically, service delivery interventions affect: (i) to whom care is provided and efforts made to reach them, (ii) by whom care is provided, (iii) where care is provided, (iv) with what information and communication and technology is care provided, and/or (v) how the quality and safety of care is improved and monitored.

### Search strategy

We adapted the search strategy from Lewin et al., who conducted a systematic review of reviews of evidence of the effectiveness of service delivery interventions in primary health care in LMICs. To adapt this strategy we removed terms pertaining specifically to reviews and specifically to primary care. We added terms to identify economic evaluations including cost-benefit analysis, cost-effectiveness analysis, cost per DALY, cost*, economic evaluation and so forth. The full search strategy is available in the Supplementary Information. We searched PUBMED, MEDLINE, EconLit, and NHS EED for studies published between 1st January 2000, when the millennium development goals were initiated, and 30th October 2016. There were no language restrictions. LMICs were defined according to the World Bank [14]. This review was initiated as part of an internal project and the protocol was submitted to and approved by the medical school but was not registered with PROSPERO. It is provided in the Supplementary Information. Each abstract was reviewed independently by two reviewers. Full text articles were obtained for all studies selected by at least one reviewer. Disagreements about inclusion were resolved through team discussion.

### Inclusion criteria

Studies were included if: (i) it was an economic evaluation that reported both incremental outcomes and incremental costs. These included studies reporting incremental cost-effectiveness ratios (ICER; e.g. cost per case cured), incremental cost-benefit ratios (e.g. cost per DALY), and expected incremental net benefit estimates (net monetary value); (ii) the study examined a service delivery intervention as defined above; (iii) the study considered only intervention(s) implemented in a LMIC, and (iv) the study was published between 1st January 2000 and 30th October 2016.

### Exclusion criteria

Evaluations reporting only cost differences, effectiveness estimates, or average costs or effects were excluded, including cost-minimisation analyses. No restrictions on language were applied.

### Data extraction

The following data were extracted from each study where reported: author(s); publication date; study date; description of intervention(s) and comparator(s); study country(ies); methods; source(s) of effectiveness evidence and their sample size(s), unit of effectiveness, mean age(s) of participants, and sex; source(s) of costs evidence; mean and median costs for treatment and comparator and their differences and 95% confidence intervals; currency; cost utility; cost benefit or cost-effectiveness ratio and 95% confidence intervals or expected net benefits; probability cost-effective; time horizon, and perspective. HS extracted the data and SW independently cross-checked the data against the original studies.

### Quality assessment

The quality of the economic evaluations was judged using the ten point Drummond checklist. [15] Each study was scored independently by two authors (SW and HS) out of a maximum of ten points. Disagreements were resolved by discussion.

### Synthesis and evaluation

Interventions were grouped according to the taxonomy in Lewin et al. (Panel 1) discussed above [7]. Estimates of ICERs or equivalent outcomes were grouped by both disease area and outcome. Permutation plots were completed for interventions judged through discussion to be similar and with similar comparators [16]. To aid comparability between results from different countries and over different time periods, ICERs, or equivalent outcomes, were converted into multiples of GDP per capita for the relevant year and country. This also permits comparability with World Health Organisation (WHO) recommendations of cost-effectiveness thresholds; less that one times GDP per capita per DALY averted is considered highly cost-effective and less than three times is considered cost-effective [17]. GDP per capita was obtained from the World Bank for the year of the relevant study in nominal, purchasing power parity terms to enable comparability between studies of different years in this metric. Where study year was not reported, publication year was used instead. A meta-analysis was not deemed appropriate for these data, as service delivery interventions are typically adapted to specific contexts, and methods and data differ widely between studies.

## Results

### Studies included and excluded

**Fig. 1:**
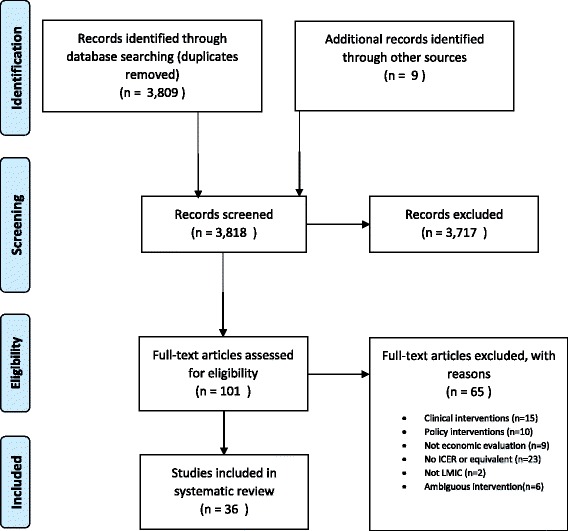
PRISMA diagram

Overall, 3818 potentially relevant abstracts were identified of which 101 studies were selected for full text review. Sixty-five studies were excluded: 15 were considered clinical interventions and 10 were considered policy (governance or finance) interventions, nine studies were not economic evaluations; an incremental ratio or net benefits could not be calculated from 23 studies, two studies were not in an LMIC country, and the remaining six did not report on a specific intervention. In total, 36 full text articles were included in the review (Fig. 1). The included studies are reported in Table 1. The clinical topics covered were HIV (ten studies), obstetric care (four studies), child health (three studies), while the rest covered other or multiple areas of care.

**Table 1:** Summary of studies included in the review

Study	Intervention(s)	Comparator	Countries	Disease area	Quality	Incremental intervention costs	Effectiveness measure	Incremental benefits	ICER	Multiples of GDP per capita per unit of effectiveness^a^
By whom care is provided										
Long et al. (2011) [18]	**Nurse led care**	Doctor led care	South Africa	HIV	7	−59 (US$ 2009)	In care and responding	+ 6 pp	− 983*	**−0.09**
Barton et al. (2013) [19]	(i) Cohort 1 (ii) Cohort 2 Nurse led care	Doctor led care	South Africa	HIV	9	(i) 103 (ii) 59 (US$ 2009)	(i) Death rate (ii) Undetectable viral load	(i) -0.4 pp. (ii) -0.5 pp.	(i) 24,500 (ii) 12,584	(i) 2.26 (ii) 1.16
Marseille et al. (2014) [26]	(i) Dedicated mobile male circumcision teams (ii) Non-dedicated, mobile integrated male circumcision	Standard care	Kenya	HIV	5	(i) 29.32 (ii) 46.20 (US$ 2009)	HIV cases averted	NR	(i) 117 (ii) 185	(i) 0.05 (ii) 0.08
Khan et al. (2002) [33]	(i) **DOTS observed by health workers**^**b**^ (ii) DOTS observed by family members (iii) DOTS delivered at health centre	DOTS without direct observation	Pakistan	TB	6	(i) 12 (ii) 0 (iii) 72 (US$ 1998)	Cure rate	(i) + 5 pp. (ii) -7 pp. (iii) -4 pp	(i) 260*^b^ (ii) 0* (iii) -1800*	(i) **0.11**^**b**^ (ii) 0 (iii) -0.73
Islam et al. (2002) [28]	**CHW provided community care**	Health facility doctor-led care	Bangladesh	TB	5	−31.8 (US$ 1997)	Cure rate	+ 2 pp	− 1590*	**−1.39**
do Prado et al. (2011) [39]	**Guardian supervised DOTS**	CHW supervised DOTS	Brazil	TB	6	− 158 (US$ NR)	Cure rate	+ 14 pp	− 1095*	**−0.08**
Prinja et al. (2014) [40]	Two auxiliary nurse midwives	Single auxiliary nurse midwife	India	Obstetric care	7	−18 (INR 2012)	ANC coverage	+ 18 pp	23,058	0.07
Gaziano et al. (2014) [29]	Providing CVD training to CHWs	Standard care	South Africa	CVD	8	6.56 (US$ 2012)	DALYs	0.0205	320	0.04
Saokaew et al. (2013) [30]	Pharmacist participated warfarin management	Standard care	Thailand	Patients receiving warfarin	7	3083 (US$ NR)	DALYs	0.79	3882	0.71
Buttorff et al. (2012) [31]	**Lay health worker task sharing**	Doctor/specialist led care	India	Mental health	8	−46 (US$ 2009)	QALYs	0.02	− 2300*	**−0.38**
Jayaraman et al. (2009) [32]	Trauma care program for lay first-responders	No program	Uganda	Emergency care	5	0.36 (US$ NR)	Death rate	NR	598	0.50
To whom care is provided										
Brooker et al. (2008) [52]	School-based helminth control program	No program	Uganda	Helminthiasis	10	0.54 (US$ 2005)	Anaemia risk	−16.7 pp	3.19	0.00
Lo et al. (2015) [53]	Community-wide mass drug administration	School-based helminth control program	Côte d’Ivoire	Helminthiasis	7	240,695 (US$ 2014) [Total]	DALYs	1443	167	0.11
Where care is provided										
Babigumira et al. (2009) [20]	(i) Mobile clinic delivered care (ii) Home based care	Facility based care	Uganda	HIV	6	(i) 1569 (ii) 2251 (US$ 2008)	QALY	(i) + 0.6 (ii) + 0.8	(i) 2615 (ii) 2814	(i) 2.27 (ii) 2.45
Babigumira et al. (2011) [21]	Pharmacy only refill program	Standard care	Uganda	HIV	8	− 135 (US$ NR)	Favourable immune response	-1 pp	13,500	10.31
Mulogo et al. (2013) [22]	**Home based testing**	Facility based testing	Uganda	HIV	3	−1.4 (US$ 2008)	Cases identified	+ 2 pp	**−3.5**	**−0.00**
Bassett et al. (2014) [23]	Mobile testing	Facility based testing	South Africa	HIV	5	100 (US$ 2012)	Life expectancy	+ 0.5 months	2400	0.33
Smith et al. (2015) [24]	Community based management: (i) ART threshold ≤200 CD4 per μL (ii) No ART threshold	Facility based management	South Africa	HIV	7	(i) 157 (ii) 293 (US$ NR)	DALYs	(i) 0.20 (ii) 0.33	(i) 22,000 (ii) 8570	(i) 3.32 (ii) 1.30
Tabana et al. (2015) [25]	Home based counselling and testing	Facility based counselling and testing	South Africa	HIV	7	4.4 (US$ 2012)	Uptake of testing	+ 21 pp	19	0.00
Chanda et al. (2011) [34]	Home management	Facility based management	Zambia	Malaria	8	2.38 (US$ 2010)	Appropriately treated	+ 57 pp	4.2	0.00
Kahn et al. (2012) [35]	**Integrated community prevention program**	Standard care	Kenya	HIV, Malaria, Diarrhoea	4	32 (US$ NR)	DALYs Costs saved	0.359 NR	**NR**	**NR**
Marseille et al. (2014) [41]	Integrated community prevention program	Standard care	70 countries	HIV, Malaria, Diarrhoea	5	26–147 (US$ NR)	DALYs	0.00–1.14	7–15,886	–
Jafar et al. (2011) [36]	(i) Home health education with trained GP (ii) Home health education only (iii) Trained GP only	Standard care	Pakistan	Blood pressure	6	(i) 3.99 (ii) 3.34 (iii) 0.65 (US$ 2007)	DALYs	NR	(i) 23 (ii) 730 (iii) 807	(i) 0.02 (ii) 0.78 (iii) 0.87
Chen et al. (2012) [37]	Volunteer orthopaedic surgery trips	Standard care	Nicaragua	Orthopaedic surgery	7	711 (US$ 2010)	DALYs	+ 1.49	352	0.23
Pitt et al. (2016) [38]	Newborn home visits	Standard care	Ghana	Obstetric care	6	0.53 (US$ 2009)	Life years saved	NR	352	0.32
Quality and safety										
Goodman et al. (2006) [42]	Drug safety training for shopkeepers	No training	Kenya	Malaria	8	0.43 (US$ 2000)	(i) Appropriate treatment for malaria (ii) DALYs	(i) 13 pp. (ii) 894 [Total]	(i) 4 (ii) 18.38	(i) 0.00 (ii) 0.01
Vella et al. (2011) [27]	Full time staff and high staff patient ratio clinic	Part time staff and low staff to patient ratio clinic	South Africa	HIV	5	8410 (US$ 2006)	Patients retained	+ 30 pp	12,271	1.23
Barasa et al. (2012) [43]	Quality improvement for children in hospitals	Standard care	Kenya	All	8	19.68 (US$ 2009)	DALYs	NR	39.8–398.3 ^c^	0.02–0.17 ^c^
Curry et al. (2013) [44]	Quality improvement for rural primary care (i) 18 month intervention period (ii) Five year follow-up	Standard care	Ethiopia	All	5	(i) 5 million (ii) 5 million (US$ NR) [Total costs]	Lives saved	(i) 134 (ii) 968 [Total]	(i) 37,313 (ii) 5071	(i) 74.60 (ii) 10.14
Broughton et al. (2011) [45]	**Quality improvement in children’s hospital**	Standard care	Nicaragua	Pneumonia	5	−14 (US$ 2010)	DALYs	0.06	**− 233**	**−0.15**
Clark et al. (2012) [46]	Quality improvement	Standard care	Sierra Leone	Emergency care	5	29,714 (US$ 2009)	Mortality risk	−6.5 pp	148	0.34
Alfonso et al. (2015) [47]	Quality improvement with voucher scheme	Standard care	Uganda	Obstetric care	8	0.41 (US$ 2010)	(i) DALYs (ii) Mortality risk	(i) 0.0014 (ii) -0.002 pp	(i) 302 (ii) 20,475	(i) 0.24 (ii) 16.51
Manasyan et al. (2011) [48]	Newborn care training in urban facilities	Standard care	Zambia	Obstetric care	6	20,223 (US$ 2005)	DALYs	NR	5.24	0.00
Prinja et al. (2016) [49]	Integrated newborn and child health program	Standard care	India	Child health	8	4.7 (US$ NR)	DALYs	NR	34.5	0.01
Information and communication technology										
Li et al. (2012) [50]	Electronic medical records	Standard care	China	All	4	NR	Net benefits ($) [Hospital level, 6 year horizon]	NR	559,025	NR
Anchala et al. (2015) [51]	Decision support system	Standard care	India	Blood pressure	9	25.79 (US$ NR)	mm Hg	−6.59	3.91	0.00

ANC = antenatal care; DALY = disability adjusted life year; DOTS = directly observed treatment; ICER = incremental cost-effectiveness ratio; NR = not reported;

pp = percentage point; QALY = quality adjusted life year

**Bold** indicates an intervention that dominates (i.e. less costly and more effective) the alternative(s)

Underlined indicates that an intervention would be considered highly cost-effective by WHO standards (< 1× GPD per capita per DALY averted)

Shading separates disease groups

Incremental costs and effects are per person unless otherwise stated

ICERs are in terms of the effectiveness unit given, e.g. cost per case cured

DALYs are reported as DALYs averted

^a^The ICER divided by GDP per capita in purchasing power parity terms for the study year

^b^The intervention was dominant over an alternative

^c^Multiple effectiveness scenarios considered

*ICERs were calculated from average figures given in the article and was not reported itself in the article

### Comparison of methods

The 36 included studies differed widely in terms of the measures of effectiveness, perspective, time horizon, and other methods. Table 2 details the methods and approaches used in the included studies. Nine studies were economic evaluations alongside a randomised trial, while the other studies were a mix of economic evaluations from observational evidence or modelling studies. The majority (21) of the included studies used basic arithmetic approaches to calculate ICERs or equivalent, the others used more complex model structures to extrapolate from trial-based or other sources of evidence. Most studies (26) reported conducting some form of sensitivity analysis although only 13 conducted probabilistic analyses.

**Table 2:** Methods used by the studies included in the review

				Benefits		Costs			
Study	Disease area	Perspective	Time horizon	Effectiveness estimate source(s)	Discount rate	Costing approach(es)^a^	Discount rate	Modelling method(s)^b^	Sensitivity analyses
By whom care is provided									
Long et al. (2011) [18]	HIV	Provider	1 yr	Observational study	NR	Bottom-up	NR	Arithmetic	Deterministic
Barton et al. (2013) [19]	HIV	Provider	1 yr	RCT	0%	Mixed	0%	Arithmetic	Deterministic
Marseille et al. (2014) [26]	HIV	Provider	20 yrs	Previous modelling study	3%	Bottom-up	NR	Arithmetic	NR
Khan et al. (2002) [33]	TB	Societal	< 1 yr	RCT	NR	Mixed	NR	NR	NR
Islam et al. (2002) [28]	TB	NR	1 yr	Observational study	NR	Bottom-up	5%	Arithmetic	NR
do Prado et al. (2011) [39]	TB	Societal	< 1 yr	Observational study	NR	Bottom-up	NR	Arithmetic	NR
Prinja et al. (2014) [40]	Obstetric care	Provider	1 yr	NR	NR	Bottom-up	3%	Arithmetic	NR
Gaziano et al. (2014) [29]	CVD	NR	3.5 yrs	Previous observational and RCT studies	NR	Bottom-up	NR	Markov model	Probabilistic and deterministic
Saokaew et al. (2013) [30]	Patients receiving warfarin	Provider + societal	Lifetime	Observational study and previous evidence	3%	Mixed	3%	Markov model	Probabilistic and deterministic
Buttorff et al. (2012) [31]	Mental health	NR	1 yr	RCT	NR	Mixed	0%	Arithmetic	Probabilistic and deterministic
Jayaraman et al. (2009) [32]	Emergency care	NR	3 yrs	Observational study	NR	NR	NR	Arithmetic/previous study	NR
To whom care is provided									
Brooker et al. (2008) [52]	Helminthiasis	Government	3 yrs	Observational study	NR	Bottom-up	3%	Arithmetic	Deterministic
Lo et al. (2015) [53]	Helminthiasis	NR	15 yrs	Previous observational studies	3%	Previous studies and assumptions	3%	Dynamic transmission model	Probabilistic and deterministic
Where care is provided									
Babigumira et al. (2009) [20]	HIV	Provider	10 yrs	Observational study	NR	Mixed	NR	Decision tree	Probabilistic and deterministic
Babigumira et al. (2011) [21]	HIV	Societal	1 yr	Assumptions	3%	Previous studies, mixed, and assumptions	3%	Decision tree and Markov model	Deterministic
Mulogo et al. (2013) [22]	HIV	Provider	NR	Observational study	NR	Bottom-up	3%	Decision tree	Deterministic
Bassett et al. (2014) [23]	HIV	Societal	2 yrs	Previous observational studies	3%	Results from previous studies	3%	Simulated patient-level Markov model (CEPAC-I)	Deterministic
Smith et al. (2015) [24]	HIV	Provider	10 yrs	Assumptions, field studies	0%	Mixed	3%	Discrete event simulation	Deterministic
Tabana et al. (2015) [25]	HIV	Provider	< 1 yr	RCT	NR	Bottom-up	3%	Arithmetic	Deterministic
Chanda et al. (2011) [34]	Malaria	Provider	NR	Observation study	NR	Bottom-up	5%	Arithmetic	NR
Kahn et al. (2012) [35]	HIV, Malaria, Diarrhoea	NR	Lifetime	Observation study	NR	Top-down	NR	Arithmetic	Probabilistic and deterministic
Marseille et al. (2014) [41]	HIV, Malaria, Diarrhoea	Provider	3 yrs	Previous observational studies	3%	Previous studies and assumptions	3%	Arithmetic	Probabilistic and deterministic
Jafar et al. (2011) [36]	Blood pressure	Societal	2 yrs	RCT	5%	Bottom-up	5%	Arithmetic	Probabilistic and deterministic
Chen et al. (2012) [37]	Orthopaedic surgery	Provider	Lifetime	Observational study	3%	Mixed	3%	Arithmetic	NR
Pitt et al. (2016) [38]	Obstetric care	Provider	1 yr	RCT	3%	Bottom-up	3%	Arithmetic	Deterministic and probabilistic
Quality and safety									
Goodman et al. (2006) [42]	Malaria	Provider	1 yr	Observational study	NR	Bottom-up	3%	Decision tree	Deterministic
Vella et al. (2011) [27]	HIV	Provider	10 yrs	Observational study	3%	Bottom-up	3%	Decision tree	Probabilistic
Barasa et al. (2012) [43]	All	Provider	1.5 yrs	RCT	3%	Mixed	3%	Arithmetic	Probabilistic and deterministic
Curry et al. (2013) [44]	All	NR	(i) 15 yrs. (ii) 5 yrs	Observational study	3%	NR	3%	Multiple models (LiST, DemProj, AIM)	Deterministic
Broughton et al. (2011) [45]	Pneumonia	Provider	2 yrs	Observational study	3%	Bottom-up	NR	Decision tree	Probabilistic
Clark et al. (2012) [46]	Emergency care	NR	< 1 yr	Observational study	NR	Bottom-up	NR	Arithmetic	NR
Alfonso et al. (2015) [47]	Obstetric care	Societal + provider	Lifetime	Observational study	3%	Bottom-up	NR	Decision tree and LiST	Deterministic
Manasyan et al. (2011) [48]	Obstetric care	NR	NR	Observational study	NR	Bottom-up	NR	Arithmetic	NR
Prinja et al. (2016) [49]	Child health	Societal + provider	15 yrs	RCT	3%	Bottom-up	3%	Decision tree	Probabilistic and deterministic
Information and communication technology									
Li et al. (2012) [50]	All	Provider	6 yrs	Observational study	10%	Bottom up and previous studies	10%	Arithmetic	Deterministic
Anchala et al. (2015) [51]	Blood pressure	Societal	1 yr	RCT	NR	Bottom up	3%	Arithmetic	Deterministic

ANC = antenatal care; DOTS = directly observed treatment; NR = not reported;

Shading separates disease groups

^a’^Bottom-up’ costing refers to any micro-costing or ‘ingredients-based’ approaches, ‘top-down’ refers to macro-costing or activity-based approaches, and ‘mixed’ is a combination of both

^b^Method of determining primary result. ‘Arithmetic’ refers to any approach that calculates ICER or equivalent using only basic arithmetic and does not use a model

As an illustration of methodological heterogeneity between studies, ten studies examined delivery interventions for HIV/AIDS in Sub-Saharan Africa (Tables 1 and 2) [18–27]. None of these studies used the same effectiveness measures. Some used health outcomes: QALYs, DALYs, ‘favourable’ immune response, life expectancy, death rate, cases averted, and undetectable viral load. The others used ‘surrogate’ endpoints: cases identified, uptake of testing, patients retained, and “in care and responding”. As shown in Table 2 eight of these ten studies took a provider or health care perspective [18–20, 22, 24–27] and two a societal perspective [21, 23]; the time horizon varied from less than one year to 20 years; and only four included cost savings contingent on reduced morbidity. This heterogeneity was reflected across all the studies included in the review rendering any further comparisons between studies and interventions inappropriate.

### Comparison of study quality

Quality scores varied widely according to the Drummond ten point checklist. Scores ranged from three to ten out of ten (Table 1). Aside from the difficulties with comparability between studies, the most common weaknesses in the included studies were the establishment of effectiveness evidence (question 3: 17/36 studies meeting the criterion) and the credible valuation of costs and consequences (question 6: 21/36 studies). Poor reporting also prevented determination of whether criteria were met. As reported in Table 2, it was not possible to establish discount rates for a number of studies (Question 7: 14/36 studies); discount rates were potentially not reported for short time horizons, but may have still been required for the valuation of capital.

### Assessment of task shifting interventions

The most widely studied types of delivery intervention were ones that altered “by whom and where” care was provided. We classified 11 and 12 of the 36 included studies in these categories, respectively. The predominant focus of these interventions was provision of typically doctor-led care by nurses or CHWs (eight studies [18, 19, 28–33]), or shifting place of care, such as providing treatment at home or in the community instead of at a facility (12 studies [20–26, 34–38]). The remaining three studies compared guardian to CHW supervised treatment for TB [39], increasing midwifery staffing, [40] and an integrated community-based programme for infectious disease (as compared to standard facility based care) [41].

**Fig. 2:**
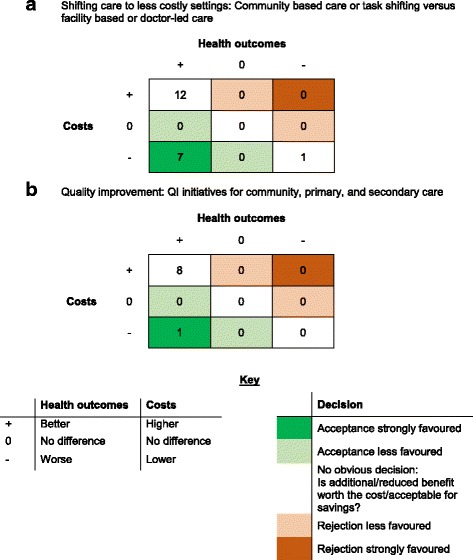
Permutation plots summarising the number of economic evaluations according to their findings. Numbers in cells represent numbers of studies. **a** Shifting care to less costly settings: Community based care or task shifting versus facility based or doctor-led care. **b** Quality improvement: QI initiatives for community, primary, and secondary care

Twenty of these 23 studies evaluated shifting care to potentially less costly settings: specifically either from doctors to nurses or CHWs, or from a facility to the home or community. Nine of these 20 reported the incremental cost per DALY averted or cost per QALY gained so that the results could be compared to WHO thresholds [20, 24, 29–31, 35–37, 41]. Seven of these nine that reported DALYs were considered highly cost-effective or cost-saving, i.e. less costly and at least as effective, when compared to WHO thresholds. A further three of the 20 studies were likely to be considered cost-effective on the basis of life years saved [23, 32, 38], and four more studies reporting other effectiveness outcomes were estimated to be cost-saving overall compared to a relevant alternative [18, 22, 28, 33]. Fig. 2(a) shows a permutation plot of the 20 studies examining shifting care to potentially less costly settings grouped by their estimated changes to costs and health outcomes and the resulting implications for decision makers. Taken together the evidence suggests that shifting tasks from doctors to CHWs, nurses, and/or from facility into the community is likely to be cost-effective and even potentially cost-saving.

### Assessment of quality improvement interventions

Nine studies reported an economic evaluation of a quality improvement initiative. Their incremental costs and benefits are summarised in Fig. 2(b) [27, 42–49]. Five of these examined quality improvement in health care facilities, all of which were considered highly cost-effective [43, 45–48]. These schemes were comprised typically of staff training and improved supervision. Medical equipment and quality assurance was also included in two of these studies [46, 47]. Beyond quality improvement initiatives in formal health care facilities, one study compared high and low staff to patient ratios, but did not find that well-staffed HIV clinics were cost-effective compared to the standard care with lower staff-patient ratios [27]. A large rural primary care quality improvement initiative in Ethiopia that implemented and improved community and primary care infrastructure, funding, and services simultaneously also failed to demonstrate cost-effectiveness [46]. However, drug safety training for drug-dispensing shopkeepers was estimated to be highly cost-effective in Uganda [42]. This latter finding reflects the results from similar, non-quality focussed initiatives included in the review but classified in other categories, such as interventions to improve the knowledge of lay trauma first-responders in Uganda [32], and providing cardio-vascular disease training to CHWs in South Africa [29]. Taken altogether there appears to be good evidence that quality improvements through training and supervision are highly cost-effective. Only four quality improvement studies appeared to take into account cost-savings to the health service contingent on reduced morbidity [43, 45, 47, 49].

### Assessment of other types of service delivery intervention

There were four further studies included in the review that examined a range of different interventions. Two considered information and communication technology interventions for hospitals, one in China [50] and one in India [51]. Two articles considered interventions expanding helminth control programs: one to schools [52] and the other to the whole community [53]. All four of these interventions were estimated to be cost-effective or cost-saving.

## Discussion

For two broad classes of intervention identified in this review the evidence suggests that they are cost-effective or potentially cost-saving. Firstly, shifting basic care from facilities to the community or the home, or from doctors to nurses or CHWs has been shown to be cost-effective across a broad range of conditions. Testing, counselling, and management of HIV at home was generally more costly but also more effective than doing so in facilities. Similarly, involving and training lay members of the community in emergency care, CVD management, and mental health all proved cost-effective. However, the task shifting programs were generally small-scale, local initiatives by researchers or healthcare providers, Further evidence is required to assess these interventions at scale. Secondly, quality improvement initiatives in community, primary, and secondary care involving staff training, supervision, and equipment upgrades were also generally considered cost-effective. However, the significant heterogeneity in the design and reporting of the economic evaluations rendered more specific quantitative comparisons of results between interventions inappropriate.

A key purpose of economic evaluation is to inform decisions. But even within the same disease area in the same region, a decision maker would have difficulty synthesising the available studies to come to an informed decision on the basis of the studies reviewed here. For example, service delivery interventions for HIV in Sub-Saharan Africa used a wide range of different outcomes, perspectives, methods, and time horizons. Without knowledge of how the results compare in terms of general cost-effectiveness measures, such as a cost per DALY, a decision maker would not be able to choose from among the many similar but ultimately mutually exclusive alternative organisational arrangements. There is a strong case for a more standardised way of conducting and reporting economic evaluations in this area, while respecting the differences between context-specific interventions. This is a key area for future research and reporting guidelines.

Lewin and colleagues studied service delivery research across LMICs ten years ago and noted a ‘dearth of evidence in low and middle income countries’ [7]. More recent reviews of service delivery interventions, such as CHW programs, [11, 12] show that the evidence base is growing in this area. Nevertheless, these recent reviews of CHW programs and the results presented here demonstrate that there is still insufficient good quality evidence to support policy makers’ decisions in this area. The subject is clearly still at an early stage of development and the time is propitious to influence the development of the field.

There is no clear line that delineates service delivery interventions from policy or clinical interventions. Rather these interventions exist on a spectrum. A clinical intervention is applied directly to a patient whereas a service delivery intervention is applied to the organisation of a clinical intervention and its delivery [5]. As such, there may be some disagreement with regard to the eligibility of studies selected for inclusion here, for example, whether the extension of helminth control programs or public health behavioural change interventions are service delivery interventions. Governance and finance arrangements were excluded as they were classed as policy interventions and concern decision makers at a different level. For example, we excluded an economic evaluation of a private sector capitation model for the public sector for diabetes in South Africa [54]. The economic evaluation of health care policy in LMICs is important for effective decision making on health care organisation and should be the subject of future research. We did not formally consider publication bias given the lack of an agreed method to do so for economic evaluations. It is possible that there is a bias towards publishing results that appear cost-effective, or that economic evaluations are undertaken only for interventions thought to be effective. This is another important area for future research.

The design and organisation of health services is a growing and important issue. The severe resource limitations in many LMICs necessitate high quality economic evaluation. Guidelines and data resources for economic evaluation in LMICs such as WHO Choice are available [55]. However, there is a high level of methodological heterogeneity between studies in LMICs and when compared to studies in high income countries. This may be due to levels of health economics research capacity or different expectations of funders, journals, or peer reviewers [56]. Previous research has also demonstrated the wide differences in results from different approaches, such as between top-down and bottom up costing approaches [57]. A key recommendation of this study is therefore standardisation of research methods and ensuring adherence to reporting guidelines. This can be achieved by funders, journals, and policy makers. Standardising outcomes may be hindered by data limitations, although DALY values for many diseases are published, such as by the Global Burden of Disease studies [58]. Other studies have emphasized the need for broad measures of general health and well-being to both capture benefits across multiple dimensions and ensure comparability between studies [59].

## Conclusions

There is a large evidence base supporting effective and cost-effective treatment of the diseases afflicting LMIC such as HIV, TB, and malaria. However, the evidence supporting the optimal configuration of services is highly limited and is an important direction for future research.

Available evidence generally supports shifting care to less costly settings, such as in the community instead of a health care facility, or task shifting from doctors to CHWs. Quality improvement initiatives were generally found to be more costly and more effective. However, there is significant heterogeneity in the literature, both in methodological approaches and in quality. This renders further comparisons difficult and limits the utility of the available evidence to decision makers. Given the severe resource limitations in LMICs, there is a pressing need for high quality economic evaluation of service delivery interventions and a need for standardisation of methods and reporting to facilitate its use to decision makers.

## Abbreviations

CHW: Community health worker
CVD: Cardiovascular disease
DALY: Disability adjusted life year
GDP: Gross domestic product
HIV: Human immunodeficiency virus
ICER: Incremental cost-effectiveness ratio
LMIC: Low and middle income country
QALY: Quality adjusted life year
TB: Tuberculosis
